# Theranostic Radioembolization: Radiation Dosimetry-Guided Treatment Planning and Delivery

**DOI:** 10.1055/s-0045-1809343

**Published:** 2025-05-23

**Authors:** Dale L. Bailey, Elizabeth J. Bernard, Richard Maher, Albert C. Goh, Yaser H. Gholami, Nick Pavlakis, Kathy P. Willowson

**Affiliations:** 1 Department of Nuclear Medicine, Royal North Shore Hospital, Sydney, Australia; 2 School of Health Sciences, Faculty of Medicine & Health, University of Sydney, Sydney, Australia; 3 Department of Medical Imaging, Royal North Shore Hospital, Sydney, Australia; 4 School of Biomedical Engineering, Faculty of Engineering, University of Sydney, Sydney, Australia; 5 Department of Medical Oncology, Royal North Shore Hospital, Sydney, Australia; 6 Institute of Medical Physics, Faculty of Science, University of Sydney, Sydney, Australia

**Keywords:** radioembolization, theranostic, hepatic malignancy, SIRT, liver-directed therapy

## Abstract

Radioembolizaton of hepatic malignancy is an accepted palliative treatment option in many subjects. The process of working up an individual for a radioembolization procedure permits pretreatment radiation dosimetry to be estimated, which is not possible with many other theranostic pairs of radionuclides. These estimates can then be used to prescribe the desired amount of radionuclide therapy (RNT), in radiation dose units of gray (Gy), to treat the cancer tissues to a desired level as well as permitting the radiation dose to the normal liver compartment to be minimized. As such, radioembolization represents an excellent example of a theranostic approach to treatment where individualization of the therapy can be highly tailored. The necessary tools are now available to implement this approach on a wider scale, which should improve outcomes for the treated individuals. The aim of this review article was to present a contemporary approach to personalized treatment planning for radioembolization and to emphasize the theranostic aspects of the process. A clinical case is presented demonstrating the potential for excellent clinical outcomes using an image-based and informed treatment plan developed by the multidisciplinary team of nuclear physicians, interventional radiologists, medical oncologists, and medical physicists.

## Radioembolization as a Theranostic Process

The recent widespread introduction of the term *theranostic* when applied to radionuclide imaging and radionuclide-based therapies implies that an imaging procedure is used to determine the suitability of the individual subject for the subsequent therapy based on the same targeting moiety, be it a radiochemical, peptide, monoclonal antibody (mAb), or some other radiolabeled vector. Common examples of theranostic radionuclide combinations (diagnostic/therapeutic) include radioiodine ( ^123^ I or ^124^ I/ ^131^ I) for thyroid conditions such as hyperthyroidism or thyroid cancer, peptides labeled with radiometals such as ^68^ Ga/ ^177^ Lu or ^64^ Cu/ ^67^ Cu for receptor targeting in neuroendocrine tumors (NETs) and prostate cancer, and longer-lived radiotracers for antibody-based therapies such as ^89^ Zr combined with ^90^ Y, ^177^ Lu, or, increasingly, α particle emitting therapeutics such as ^225^ Ac and ^212^ Pb. There are now a multitude of emerging biotechnology companies trying to develop new theranostic agents 1 given their efficacy and ease of delivery as a systemic treatment, usually in the setting of metastatic cancer.

Radioembolization, a form of targeted radionuclide therapy (TRT), was first introduced in the 1960s for the intra-arterial delivery of radiation attached to microparticles to treat liver and pancreatic malignancies. The early studies used ceramic microspheres labeled with yttrium-90 ( ^90^ Y: t _½_ = 64 hours) to deliver a therapeutic radiation dose to the neoplastic tissues. The early microspheres were in the range of 50 ± 10 μm in diameter. 2 Yttrium-90 is still the most commonly used radionuclide for what is now referred to as selective internal radionuclide therapy (SIRT), although holmium-166 ( ^166^ Ho: t _½_ = 26.8 hours) has been proposed more recently. 3 Radioembolization has traditionally been used as a palliative treatment but as knowledge and experience with dosimetry of ^90^ Y and dose/response effects are established in a robust, reproducible methodology, new clinical aims such as segmentectomy for oligometastatic disease are being pursued for potential curative intent.

In this review article, we present a contemporary approach to personalizing the delivery of a radioembolic therapy and emphasize the theranostic nature of the workup, treatment planning, delivery, and subsequent assessment of efficacy.

The typical process for the workup of a subject for liver-directed therapy (LDT) using radioembolization includes a surrogate embolization procedure using a short-lived radionuclide attached to a radiolabeled microsphere, which can be imaged in the days before the planned treatment date to check several aspects of the procedure, including:

- whether the vascular anatomy of the subject allows the interventional radiologist to deliver the microspheres to the target tissues;
- whether the malignant tissues take up the microspheres to a sufficient degree to be able to deliver a sufficient dose of radiation;
- whether there is any off-target deposition of the microspheres that could lead to complications or potentially life-threatening damage to normal tissues; and
- the degree of arterial-venous shunting present, which could see the therapeutic microspheres bypassing the targeted tissue and hence lodging in the vascular bed of the first organ they encounter, that is, the lungs.

It is most common to use [ ^99m^ Tc]-labeled microspheres for this pretreatment evaluation, in spite of the fact that the microspheres used for the workup evaluation and the subsequent treatment are not the same and could, in fact, be quite different physically in size and shape. 4

Radioembolization is often not mentioned when discussing theranostic procedures, possibly because its mechanism of action is more akin to a medical device (an embolic microsphere) rather than the molecularly guided, receptor-targeted systemic therapies that have seen the rapid rise of theranostic agents, but also possibly because it predated the molecular approaches in the same way that radioiodine is often neglected as a theranostic in spite of its use as such for over 75 years. 5 However, in many ways radioembolization is an exemplar of the theranostic approach as it allows pretreatment screening as well as providing the ability for an individualized amount of therapeutic radionuclide to be calculated from pretreatment whole organ or voxel-based dosimetry. This is because the distribution of the microspheres, once introduced into the body, does not change over time remaining static unlike the peptide- or mAb-based therapies, which are constantly changing biodistribution over time, as well as the short half-life of the imaging component of the therapeutic pair (e.g., ^68^ Ga: t _½_ = 68 minutes, ^18^ F: t _½_ = 109.8 minutes) often being too short to provide radiation dose estimates for the subsequent treatment (e.g., ^177^ Lu: t _½_ = 6.7 days).

In our institution today the practice of radioembolization for liver malignancy involves a workup sequence that evaluates the target lesion vascularity and distribution with triple-phase computed tomography (CT) angiography, an assessment of hepatic function using [ ^99m^ Tc]-mebrofenin, 6 similar to what is done for the kidneys for peptide therapies to assess pretreatment renal glomerular filtration rate, functional imaging with an appropriate radiopharmaceutical (e.g., [ ^18^ F]-fluorodeoxyglucose [FDG], [ ^68^ Ga]-prostate-specific membrane antigen, or [ ^68^ Ga]-dodecanetetraacetic acid [DOTA]-octreotate), and finally a ^99m^ Tc-based microsphere embolization mimicking the procedure that will be used in the treatment for treatment planning from which the radiation dose is calculated for both the targeted malignant lesions as well as the normal liver, allowing a highly informed prescription of the amount of SIRT required to achieve the optimum outcome by maximizing the dose to the target lesions while minimizing the dose to nontarget tissues.

## Radiobiological Considerations in ^90^ Y Radioembolization

A multitude of factors are relevant when considering the biological response of human tissues to any irradiation. These include the type of radiation (X-ray, γ, β ^-^ , β ^+^ , α ^2+^ , e ^-^ , Auger e ^-^ , etc.), the energy of the emitted radiation, duration of irradiation (exposure time or half-life of radionuclide), and dose rate as well as biological parameters such as biological sensitivity to the type of radiation, repair mechanisms after single or double strand deoxyribonucleic acid (DNA) breaks, production of chemical radicals within the targeted cell, proliferation rate of the cells, hypoxic status, and the interaction point of the radiation with respect to critical structures within the cell such as the nucleus and mitochondria.

A frequent misconception in radiation dosimetry considerations is that the amount of biological damage from 1 gray (Gy) of external beam radiation therapy (EBRT) using megavoltage (MV) X-rays delivered at a dose rate of 1 to 20 Gy/min is equivalent to 1 Gy of TRT using a slowly decaying radionuclide, which is emitting particulate radiation (i.e., α ^2+^ , β ^-^ , β ^+^ , Auger e ^-^ ) at a dose rate of 0.002 to 0.02 Gy/min. A group in Oxford (United Kingdom) studied the differences in cell survival between EBRT and ^90^ Y irradiation in colorectal cancer cell lines using a clonogenic assay. They found that ^90^ Y and EBRT cause markedly different effects for the same total dose delivered that could be ascribed to the continuous low dose rate of ^90^ Y radiation. 7 They then applied these laboratory-based results to a series of patients treated with ^90^ Y-SIRT. They found that the α/β ratios of the linear-quadratic (LQ) model of cell survival 8 for both normal and tumor tissues for the same total absorbed dose were different between EBRT and ^90^ Y and that a formula relating the biological effective dose (BED) for different radiation modalities was necessary. Their results suggest that the BED _90Y_ was equivalent to approximately 0.6 times the BED _EBRT_ . 9 That is, a calculated dose of 50 Gy from ^90^ Y is more likely to have the same effect as 30 Gy of EBRT. This may well be a contributing factor to why radioembolization treatments are often felt to “undertreat” the malignancy based on the calculated dose delivered in Gy alone.

In our institution, we have also looked at clinical biological parameters that might be able to predict response and hence modify the treatment plan accordingly. 10 . In any TRT the initial dose rate decreases exponentially over time and there exists a critical time ( *T*_crit_ ), associated with a critical dose rate ( *R*_crit_ ), where permanent DNA damage (the probability of causing double strand breaks) effectively becomes exceedingly low due to the rate of enzymatic DNA repair. Single strand breaks of DNA, which is the dominant initial damage from β ^-^ radiation, have been reported to be 90% repaired after 1 hour by enzymatic activity. 11 Both *T*_crit_ and *R*_crit_ are dependent on the α parameter in the LQ model (radiosensitivity of the tumor cell), the radionuclide half-life, initial dose rate, and the cell repopulation time ( *T*_p_ ). 12 The other key radiobiological parameter that adversely affects the local tumor control and/or survival is the so-called “kick-off” time ( *T*_k_ ). 13 Previous studies have shown that for certain cancer types, particularly highly proliferating cancers with *T*_p_ ranging from a few days to a few weeks, there exists a short period of time after the start of radiotherapy before the tumor starts to grow more rapidly than prior to irradiation and this is referred to as the “kick-off time.” Since the dose rate in RNT is mono- or bi-exponentially decreasing with time and the treatment time is usually long (e.g., weeks to months), the *T*_k_ can significantly impact the BED and treatment outcome (e.g., tumor control probability, or TCP). 12 In our retrospective study of SIRT patients it was estimated that the LQ model parameter values for α and α/β were in the range of approximately 0.001 to 1 Gy ^−1^ and approximately 1 to 49 Gy, respectively. 10 We demonstrated that the time factors, *T*_p_ , *T*_k_ , and *T*_crit_ , were the key parameters when evaluating hepatic lesional response to [ ^90^ Y]SIR-Spheres treatment. Patients with cholangiocarcinoma have been shown to have the longest average *T*_p_ (≈236 ± 67 days), highest TCP (≈53% ± 17%), and total liver lesion glycolysis response (ΔTLG _liver_ ≈ −64%), while patients with hepatic metastatic colorectal cancer have the shortest average *T*_p_ (≈129 ± 19 days), lowest TCP (≈28% ± 13%), and ΔTLG _liver_ ≈ −8%, respectively. A trial is currently underway in our institution to acquire two FDG positron emission tomography (PET) scans in the 4 weeks prior to SIRT to better evaluate the rate of progression

## Understanding the Dose–Response Relationship

Knowledge of target and threshold doses to inform and optimize treatment can only be obtained through posttreatment dosimetry, which requires direct imaging of the ^90^ Y microspheres in vivo. In recent years, this has moved from qualitative, poor-quality Bremsstrahlung single-photon emission computed tomography (SPECT) imaging to fully quantitative PET data. 14 While ^90^ Y PET provides a quantitative reconstruction, and hence dosimetry, 15 the images are typically of poor quality and require at least 20 minutes acquisition time centered on the liver even for the most sensitive total-body PET scanners. 16 Aside from access to the PET scanner, another challenge is the difficulty in performing independent calibration or quantitative confirmation on the scanner as this typically requires ^90^ Y citrate (or solution) rather than the microspheres, which rapidly settle in solution making phantom imaging challenging.

Despite these obstacles ^90^ Y PET has now become a mainstream approach for post-SIRT confirmation and comparison for response to treatment and survival is shaping our perception of targets, thresholds, and significant prognostic factors. It is well recognized that differing pathologies will demonstrate different dose requirements, a fact that includes the underlying variations in tumor doubling time, radiation sensitivity, and factors such as mutation status. Such processes are further complicated by the inability of SIRT to produce a uniform radiation field, which is instead at the mercy of vascular anatomy, flow, and accessibility. The relationship between dose and response is further complicated by prior treatment and patient clinical history, both of which may have impacted the patient's liver parenchyma and its ability to either cope with radiation insult or shift function to spared regions together with mutation and radioresistance of disease. Indeed, evidence suggests that treating bilobar disease in a sequential manner does allow liver function to shift and enhance in the spared lobe suggesting that sequential treatment may be more beneficial for patients with whole liver disease allowing higher doses to be achieved in a safe framework. 17 Overall, the relationship between radiation dose from ^90^ Y-SIRT and measured response is a particularly complex one to understand.

Examples of prognostic measures that may prove useful when predicting both response to SIRT as well as the patient's ability to withstand potentially high doses to normal parenchyma include the albumin-bilirubin score, 18 aspartate transaminase-to-platelet ratio, international normalized ratio, the use of personalized dosimetry and higher absorbed dose, 19 and the impact of prior treatment and presence of bilobar disease. 20 An active area of research when considering enhancing the effect of SIRT is that of SIRT in combination with other treatments such as radiosensitizers and targeted EBRT for regions inaccessible or nonresponding to SIRT, such as stereotactic body radiation therapy. An area of particular interest with promising results is that of immunotherapy used in combination with SIRT for hepatocellular carcinoma to enhance outcomes (overall survival and progression-free survival). 21 22

## From Naive to Well-Informed SIRT Prescribing

Radionuclide therapies delivered over many years by nuclear medicine practitioners have tended to follow a “one size fits all” approach to prescribing. Radioiodine ( ^131^ I) treatment for thyroid cancer after thyroidectomy, so-called adjuvant ablation, is one example: the amount of radioactive iodine prescribed rarely varies from a couple of standard values based on perceived risk and aggressiveness of the subject's type of thyroid cancer. 23 Most people in our institution are prescribed 4 GBq of ^131^ I after surgery but that can be decreased to 2 GBq in patients deemed to be at greater risk of long-term complications (such as being relatively younger) or can be increased in particularly aggressive forms of cancer to 6 GBq. Other factors such as the patient's height and weight or burden of disease are not considered relevant. This is almost diametrically opposed to modern EBRT treatment planning, which involves detailed tissue segmentation on cross-sectional imaging, defining the tumor and treatment volumes, and even enhancing dose delivery at some locations thought to be at higher risk for future recurrence (“boost dose”). A corollary of the “one size fits all” approach is that invariably some subjects will be underdosed and could have been given more therapy to increase the chances of a good response while others are overdosed and may develop side effects either acutely or at a later time.

Radioembolization of hepatic malignancies began in the modern era with the work of Gray et al. 24 He developed SIR-Spheres (Sirtex Medical, Woburn, Massachusetts, United States) (Sirtex Medical SIR-Spheres resin microspheres is the only regulator-approved product for radioembolization available in Australia, and thus the only product we have experience with, and so will be used for all examples. Doing so is not intended to suggest any implications for treatment or outcomes over other products), which are resin-based, physiologically inert microspheres measuring approximately 30 ± 10 μm in diameter that are impregnated with ^90^ Y. A vial of SIR-Spheres at calibration time contains 3 GBq of ^90^ Y in approximately 44 × 10 ^6^ spheres, or roughly 50 Bq of ^90^ Y per microsphere. 25 The ^90^ Y β ^-^ particles have a mean range in tissue of 2.5 mm and a maximum range of 10 to 11 mm. This path length is necessary as the particles will lodge in the capillary bed of the vasculature of the tumor rather than in the tumor cells as for most other molecular TRTs. The microspheres are delivered by branches of the hepatic arterial supply to the liver rather than the portal venous circulation as the malignant tissue relies on the arterial blood supply in preference to the portal venous to satisfy its oxygen demands. Therefore, delivering the microspheres via the arterial circulation preferentially targets the tumor tissues with relative sparing of the normal liver parenchyma, which only obtains approximately 20% of its blood supply from the arterial vasculature.

Starting with the simplest formula based on the medical internal radiation dose (MIRD) formalism, the estimated radiation dose to the treated compartment ( *D _c_* ) for a given amount of ^90^ Y radioactivity ( *A _c_* ) can be calculated as:



where *M _c_* is the mass of the perfused liver volume. 26 27 It can be seen that for a typical liver mass of approximately 1.5 kg that a 1.5 GBq ^90^ Y injection will deliver a dose of approximately 50 Gy to the targeted tissue. This simple model assumes that ^90^ Y distributes uniformly within the tumor tissue and normal hepatic parenchyma to provide an evenly distributed absorbed dose. The SIR-Spheres Package Insert 28 provides two further tools that use slightly more information in determining the amount of ^90^ Y microspheres to deliver—the body surface area (BSA) model and the partition model. The BSA method varies the amount of ^90^ Y prescribed according to the size of the patient and the size of the tumor within the liver and is used when treating a single liver lobe or the entire liver. A modified version of the BSA method can be used in patients who receive lobar or segmental treatment with ^90^ Y microspheres as the prescribed radioactivity must be reduced in accordance with the size of the fraction of the liver being treated, as expressed in:



where *V* is the volume (in cm ^3^ ) of the tumor ( *t* ), treatment compartment ( *c* ), and liver ( *l* ), respectively. It will be clear to the informed reader that this equation is only an empirical one as the answer, in units of radioactivity in GBq, is calculated from an equation, which only contains units of volume and mass.

The partition model 29 30 focuses on the tolerable radiation dose to lung and normal liver, at a level specified in the calculation by the user, rather than the dose to the tumor(s). The initial amount of radioactivity is calculated based on the desired dose to the tumor ( *D _t_ )* from:



where *M _n_* and *M _t_* are the masses of the normal (nontumoral) liver and tumor, respectively, *r* is the target:normal liver ratio of the average radioactivity concentration in the tumor compared with the nontumoral liver, and *L* is the lung shunt fraction, that is, the ratio of the [ ^99m^ Tc]-macroaggregated albumin (MAA) total counts in the lungs compared with the total counts in the lungs plus the normal liver. From this initial calculation the radiation dose to the lungs can be calculated based on the lung shunt fraction and assuming a lung mass of 1,000 g using the MIRD approach and then the amount of ^90^ Y can be adjusted (either up or down) based on the estimated radiation dose to the lungs. 29 In this case, the dose received by the tumor has no upper limit. The partition model is used where the tumor mass is a discrete area within the liver. For further information on these calculations the reader is referred to the recent review by Kim et al 25 and the SIR-Spheres Package Insert. 28 Subsequent to these calculations, a reduction in the amount of ^90^ Y prescribed may be considered when any of the following conditions are met:

- heavy pretreatment with systemic chemotherapy;
- any significant hepatic functional loss due to previous LDT;
- prior hepatic surgery or radiotherapy;
- high (> 10%) lung shunt fraction; and
- cirrhosis.

All of the dosimetry methods discussed above use little spatial information and distribution of the pretreatment workup embolization procedure using [ ^99m^ Tc]MAA apart from the calculation of target:normal ( *r* ) ratio in the partition model. A more sophisticated approach is to use the images from the workup SPECT imaging and calculate the spatial distribution of the absorbed dose at the voxel level based on the perfused liver volume in relation to the tumors defined on CT or functional PET imaging (e.g., [ ^18^ F]FDG PET in most solid tumors or [ ^68^ Ga]DOTA-octreotate in NETs). Voxelized dosimetry has distinct advantages over the simplified models discussed above. Since each voxel is treated as an individual compartment, there is no limit to the volume of interest the user can define to determine dose statistics as the resulting output of the process is an image in units of Gy. This is particularly valuable when multiple tumors are present, allowing each to be interrogated individually. Voxelized dosimetry also allows more sophisticated dose parameters to be reported as opposed to just the average dose across a volume, including parameters, which have demonstrated a superior predictive relationship to response in EBRT, such as the fraction of the treatment volume that will receive at least 50 Gy (V50) or the minimum dose to 70% of the treatment volume (D70). 17 While there is some debate about the choice of a dose-kernel convolution approach versus the simplified approach of the 31 “local deposition method” to generate voxelized dose maps, both are considered to represent the gold standard approach to SIRT dosimetry. Indeed, studies have demonstrated the efficacy of personalized versus standard dosimetry in treatment outcomes 32 as well as its potential cost-effectiveness. 33 The improved flexibility and accuracy of such models does, however, come at a cost where specialized software, in-house expertise, and extensive dosimetrist time are all required.

In-house patient-specific dosimetry is becoming more feasible with a broader range of both vendor-specific and vendor-neutral platforms offering both MAA-predictive and ^90^ Y posttreatment dosimetry. While this is by no means an exhaustive list, examples of software that offer SIRT-specific dosimetry include MIM SurePlan (MIM Software Inc., Cleveland, Ohio, United States), RapidSphere (Varian Medical Systems, Atlanta, Georgia, United States), Planet Dose (DOSIsoft SA, Cachan, France), Simplicit ^90^ Y (Boston Scientific, Marlborough, Massachusetts, United States [specific for glass microspheres]), Hermia Voxel Dosimetry (Hermes Medical Solutions, Stockholm, Sweden), Torch (Voximetry, Madison, Wisconsin, United States), as well as freeware options for planning, such as the MIRDy90 platform. 34

In recent years, the publication of recommended dose targets and safety thresholds 27 has highlighted both the increasing acceptance of dosimetry-guided SIRT and its expanding role in theranostics, despite recognition that the radiobiology processes involved are yet to be fully understood. Treatment intent and the ability to tolerate some loss in liver function play a large role in interpreting the absorbed dose, which is acceptable to normal liver parenchyma, and which must in turn be considered in light of patient-specific clinical factors. While early work suggested a dose of at least 50 Gy to tumors is likely to result in a significant metabolic response, 35 this has subsequently been revised to 100 to 150 Gy 27 with recognition that targeting nonuniformity, pathology, and other clinical factors will also determine dose effectiveness. More recent data even suggests that tumor dose should ideally exceed 250 Gy. 36 It is likely that as our understanding of radiobiology improves alongside our ability to utilize software to generate more suitable dose parameters (e.g., TCP, BED, etc.), these targets and thresholds will be further refined, in particular, when it comes to tolerable dose in normal liver, which is typically the dose-limiting factor when planning treatment.

As for all theranostic approaches, imaging plays a vital part especially in radioembolization. It is used in both pre- and posttreatment evaluation. The verification of the SIRT implantation distribution can be readily imaged quantitatively with today's PET devices, in spite of the exceedingly low positron emission fraction. 15 When PET is not available, Bremsstrahlung SPECT, while not quantitative in an absolute sense, can be used if dedicated correction methods (assumed amount of ^90^ Y administered, etc.) are available. 37 PET imaging, however, remains the preferred option.

## An Ideal Patient Workflow

**Fig. 1:**
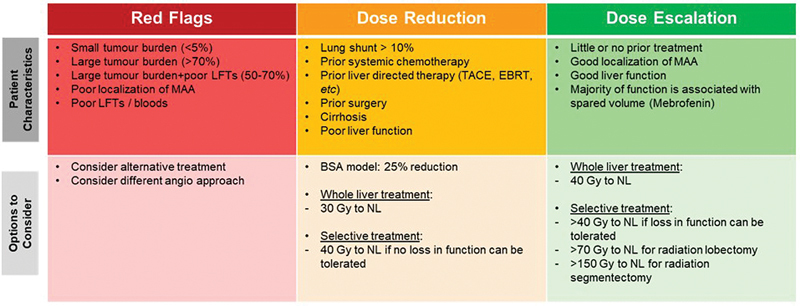
The authors' reference for categories of risk and action options when considering an individual selective internal radionuclide therapy (SIRT) treatment. BSA, body surface area; EBRT, external beam radiation therapy; Gy, gray (of radiation); LFT, liver function test (blood and biochemistry); MAA, macroaggregated albumin ([ ^99m^ Tc]MAA; NL, normal liver; TACE, transarterial chemoembolization.

While the typical patient workflow varies greatly between sites depending largely on access to resources, Table 1 represents what might be considered an ideal workflow in terms of appropriate timelines, imaging for guidance, and confirmation of dose delivery, as well as dosimetry for optimized treatment and post-SIRT management. In terms of applying and interpreting dosimetry considering the current recommendations for toxicity thresholds and clinical factors, Fig. 1 is a useful guide that we have developed. It should be noted that while target lesion doses play a role in dosimetry and prescribing activity for SIRT, it is generally the normal liver dose that carries more weight, depending of course on the targeting ability of disease preferentially over normal parenchyma.

**Table 1:** Indicative timing and procedures in the SIRT workup in the authors' institution in 2024

Time	Procedure	Comment
	MDT discussion to determine if SIRT is the most effective treatment option	Typically involves the interventional radiologist, nuclear physician, gastroenterologist, hepatologist, medical oncologist
Week –4 to week –1	Triple-phase contrast-enhanced CT of abdomen	Map vasculature and access for SIRT as well as to provide accurate volumes for segmentation
Week –4 to week –1	Diagnostic PET/CT to map tumors	Typically, FDG to determine extent of metabolically active tumor for treatment and exclude necrotic regions from consideration. [ ^68^ Ga]DOTATATE PET may be recommended in addition to FDG for NET patients to inform full extent of disease
Week –4 to week –1	[ ^99m^ Tc]Mebrofenin dynamic and SPECT/CT to map regional liver function	Particularly useful for lobar or selective treatment to determine function associated with treatment volume and hence guide dose boost or dose reduction
Day –7 to day –4	[ ^99m^ Tc]MAA liver-lung shunt study with SPECT/CT to assess lung shunt and correct targeting in liver	Data used for predictive dosimetry, either voxel-based (used to derive dose map) or partition model-based (used to derive tumor-normal liver ratio). Ideally, MDT between physicist, Nuclear physician, and IR to determine treatment approach and interpretation of dosimetry for prescribed dose. Ideally, derive lung dose as opposed to shunt as a percentage
Day 0	SIRT administration	
≤ +24 hours	^90^ Y PET or Bremsstrahlung SPECT imaging	Quantitative imaging with PET. Derived dosimetry of dose to normal liver and lesions may act as early indicator of need for prophylaxis (if high dose exceeded), likelihood of response (from individual lesion doses), and guidance for future patient therapy (based on normal liver dose)
Week +8 to +12	Follow-up PET/CT and diagnostic CT and [ ^99m^ Tc]Mebrofenin	To assess response and guide further management. In particular for patients receiving sequential treatment, follow-up can indicate whether further treatment is likely to be successful. Follow-up analysis of regional distribution of liver function can also indicate the effects of radiation dose to normal liver parenchyma and how this may have impacted global liver function

Abbreviations: CT, computed tomography; FDG, [ ^18^ F]fluorodeoxyglucose; IR, interventional radiologist; MAA, macroaggregated albumin (a microsphere); MDT, multidisciplinary team; NET, neuroendocrine tumor, PET, positron emission tomography; SIRT, selective internal radionuclide therapy; SPECT, single-photon emission computed tomography.

## Case Example

Finally, a case example is included of a 76-year-old female who was diagnosed 2 years previously with thymic carcinoma. She was referred for consideration of SIRT due to hepatic metastases. She had been previously treated with first-line (doxorubicin/cisplatin/cyclophosphamide) and second-line (carboplatin/paclitaxel) chemotherapy, which she completed approximately 9 months prior to the SIRT with only a partial response. She was subsequently referred for consideration of liver-directed treatment using ^90^ Y SIRT.

**Fig. 2:**
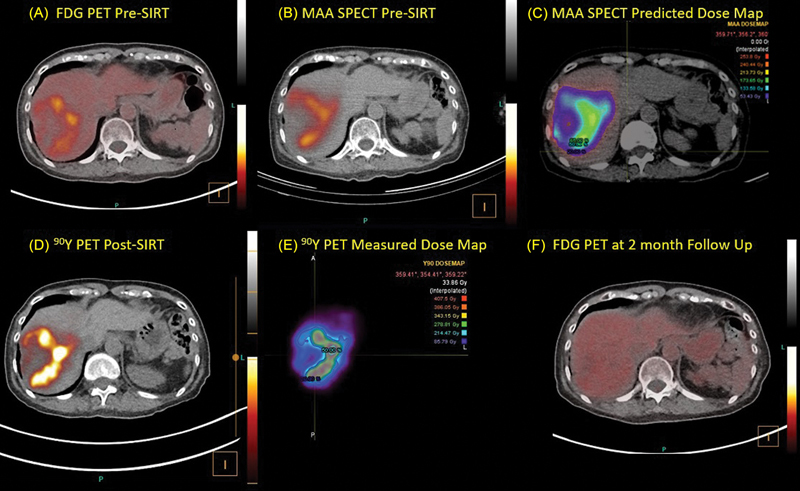
( **A** ) [ ^18^ F]-Fluorodeoxyglucose (FDG) positron emission tomography (PET) pre-selective internal radionuclide therapy (SIRT) demonstrating a large lesion in the right lobe of the liver with a highly metabolic rim surrounding a central region largely devoid of any glucose metabolism. ( **B** ) Macroaggregated albumin (MAA) single-photon emission computed tomography (SPECT) pretreatment workup scan through the same section of the liver. ( **C** ) The predicted dose map derived from the MAA SPECT scan in units of Gy. ( **D** ) Post-SIRT ^90^ Y PET demonstrating the distribution of the SIR-Spheres and ( **E** ) the corresponding measured dose map. ( **F** ) A follow-up FDG PET scan approximately 2 months after the SIRT demonstrates a complete metabolic response.

The pretreatment planning and posttreatment confirmation imaging and dosimetry for this case are shown in Fig. 2 . Fig. 2A shows a section through an [ ^18^ F]FDG PET/CT scan of the liver displaying the dominant large hypermetabolic mass in the right lobe of the liver (segment 8) with a central nonmetabolic region. Smaller metastatic deposits were also noted in liver segments 2 and 6 and in the lungs.

The amount of [ ^90^ Y]SIR-Spheres required was calculated with the three methods available (modified BSA, partition model, voxel-based dosimetry). The modified BSA calculation of the prescribed ^90^ Y amount was 1.16 GBq based on a tumor volume of 227 mL in the target treatment volume of 795 mL.

Total liver volume was measured to be 1,110 mL. Due to the functional liver [ ^99m^ Tc]mebrofenin study demonstrating borderline abnormal hepatic function (9.5%/min liver uptake rate; normal value > 11%/min), the decision was made to limit the dose to normal liver in the right lobe to less than 40 Gy while aiming to deliver 120 Gy to the lesion. Fig. 2B shows the distribution of the [ ^99m^ Tc]MAA in the pretreatment workup SPECT scan at the corresponding location for the segment 8 lesion. The partition model calculated that the normal liver compartment in the treatment volume would receive 40 Gy from 1.08 GBq of ^90^ Y. Fig. 2C shows the estimated dose map (in Gy) (Sureplan LiverY90, MIM, Cleveland, Ohio, United States) derived from the [ ^99m^ Tc]MAA scan. Volumes of interest corresponding to the areas of high uptake on the FDG PET scan were defined by thresholding the FDG PET images and were transferred to the MAA-derived dose map. This demonstrated that there was very good localization of perfusion in areas of high metabolic uptake and that 1.2 GBq of ^90^ Y would deliver an average dose of 120 Gy to the target lesion with less than 30 Gy to normal liver.

On the day of SIRT, a catheter was inserted via the right femoral artery to provide access to the right hepatic artery. During the administration, however, back-pressure was experienced in the catheter line and the full amount of [ ^90^ Y]SIR-Spheres was unable to be delivered. The quantitative ^90^ Y PET scan the following day demonstrated that only 980 MBq of the 1.2 GBq prescribed had been administered during the procedure.

**Fig. 3:**
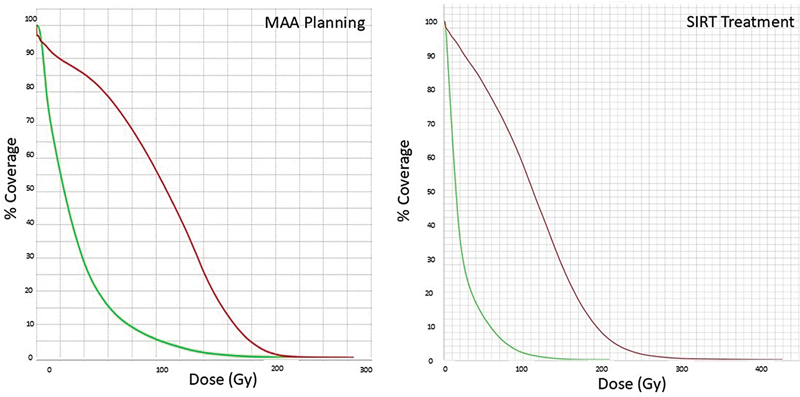
Dose-volume histograms (DVHs) from the planning macroaggregated albumin (MAA) single-photon emission computed tomography (SPECT) (left) and post-selective internal radionuclide therapy (SIRT) ^90^ Y positron emission tomography (PET) (right) distribution demonstrate a large dose of SIRT delivered to the tumor (red line) with an acceptably low level of delivery to the normal liver (green line). In this example the planning scan gave a good prediction of the subsequent SIRT distribution.

Fig. 2D shows the [ ^90^ Y]SIR-Spheres distribution after the treatment demonstrating good localization and the corresponding dose map ( Fig. 2E ) showed that the lesion received up to 400 Gy at the individual voxel level. The ^90^ Y dose maps showed a mean lesion dose of 112 Gy and a mean dose to normal liver of 23 Gy, thereby almost achieving the intended dose criteria, which would likely have been satisfied if the entire prescribed amount of [ ^90^ Y]SIR-Spheres had been able to be delivered. The dose-volume histograms ( Fig. 3 ) for both the planning [ ^99m^ Tc]MAA SPECT scan and the subsequent post-SIRT ^90^ Y PET scan showed excellent targeting of the lesion relative to the normal liver. The planning scan was a good indication of the treatment distribution. The follow-up FDG PET scan acquired approximately 8 weeks after the SIRT procedure ( Fig. 2F ) demonstrated a complete metabolic response. The follow-up post-SIRT [ ^99m^ Tc]mebrofenin study demonstrated slightly improved hepatic function of 11.0%/min liver uptake rate.

## Conclusion

SIRT for liver neoplasia involves an invasive workup procedure mimicking the interventional treatment as well as several ancillary scans. This is followed, if the patient is suitable, with the SIRT procedure to treat the patient's liver disease. As such, this should be regarded as a theranostic approach. In fact, using the methods described in this report to individualize the dose and prescribe a treatment measured in radiation dose units (Gy), rather than in units of administered radioactivity (MBq), it can be argued that the SIRT procedure is, in fact, more advanced as a theranostic than peptide receptor radionuclide therapy (PRRT) approaches using radiolabeled peptides targeting cancer cell-based receptors, which generally employs a “one size fits all” standardized dosing regimen. The fixed posttreatment distribution of the ^90^ Y microspheres makes calculation of radiation dose delivered simpler than for PRRT approaches as the biodistribution does not change over time and therefore the organ absorbed radiation dose simply follows the physical decay of the treating radionuclide. The field of radioembolization and personalized dosimetry has made great advances over the past decade and is now moving toward a precision-based individualized approach to improve outcomes as more is learnt about the radiobiology of different cancers of the liver.

## Summary: Pros and Cons of Radioembolization

Finally, Table 2 presents a summary of the pros and cons today of using radioembolization. With increasing experience of using voxel-based approaches and improvements in software capability and workflow, including using advanced processing tools to aid laborious tasks such as organ segmentation, the SIRT procedure should find increasing utility with better outcomes.

**Table 2:** Advantages and challenges associated with SIRT in the clinic

	Advantages	Challenges
Procedure	Often only performed once or twice if sequential lobar treatment offered. Minimal stay in hospital. Minimal side effects	Invasive procedure
Treatment options	Can be very selective to spare healthy liver parenchyma. Good option for patient where extrahepatic disease is well controlled on other interventional drugs but liver is progressing. Sequential lobar treatments can offer a chance to preserve/shift liver function	Some patients may not be suitable candidates for treatment due to inaccessibility of tumor blood supply, shunting, disease burden, or poorly functioning liver
Patient-specific dose planning available	Allows for a patient-specific optimized treatment. Dosimetry simplified due to lack of redistribution. Pathology-specific guidelines available to increase dose prescription confidence and likelihood of response	Software can be costly and requires time and expertise. The planning product ( ^99m^ Tc-MAA) differs in some respects from the therapeutic product ( ^90^ Y-microspheres) which may potentially lead to differences in distribution
Dose prescription	A range of models of varying complexity are available for dose prescription. Accompanying tests and imaging are available to guide and indicate appropriate dose-reduction or dose-boosting	Freely available models tend to be of a more simple nature and may not compare readily to patient-specific dosimetry-based prescription. Prescribed activity is often limited by dose to normal liver which may preclude an effective treatment dose from reaching tumors.
Dose heterogeneity	Allows for tolerance of high average dose to volumes of normal liver due to sparing of cells	Nonuniform dose across lesions may lead to areas of disease being undertreated and nonresponsive.
Imaging	Both workup and posttreatment imaging can be performed for confirmation of targeting and dose delivered	Image quantification should be used. Non-PET facilities may struggle to produce posttreatment ^90^ Y images.

Abbreviations: MAA, macroaggregated albumin; PET, positron emission tomography.
